# IL-27 induces an IFN-like signature in murine macrophages which in turn modulate colonic epithelium

**DOI:** 10.3389/fimmu.2023.1021824

**Published:** 2023-04-20

**Authors:** Caroline Andrews, Mairi H. McLean, Julie A. Hixon, Sergio M. Pontejo, Tregei Starr, Courtney Malo, Margaret Cam, Lisa Ridnour, Heather Hickman, Olivia Steele-Mortimer, David A. Wink, Howard A. Young, Daniel W. McVicar, Wenqing Li, Scott K. Durum

**Affiliations:** ^1^ Laboratory of Cancer Innovation, Center for Cancer Research, National Cancer Institute, National Institutes of Health, Frederick, MD, United States; ^2^ Division of Molecular and Clinical Medicine, School of Medicine, University of Dundee, Dundee, United Kingdom; ^3^ Laboratory of Molecular Immunology, National Institute of Allergy and Infectious Diseases, National Institutes of Health, Bethesda, MD, United States; ^4^ Rocky Mountain Laboratories, National Institute of Allergy and Infectious Diseases, National Institutes of Health, Hamilton, MT, United States; ^5^ Viral Immunity and Pathogenesis Unit, Laboratory of Clinical Immunology and Microbiology, National Institute of Allergy and Infectious Diseases, National Institutes of Health, Bethesda, MD, United States; ^6^ Center for Cancer Research Collaborative Bioinformatics Resource, National Cancer Institute, National Institutes of Health, Bethesda, MD, United States

**Keywords:** IL-27 cytokine, IBD - inflammatory bowel disease, macrophages, IFN signature, colonoid

## Abstract

Mucosal delivery of IL-27 has been shown to have a therapeutic benefit in murine models of inflammatory bowel disease (IBD). The IL-27 effect was associated with phosphorylated STAT1 (pSTAT1), a product of IL27 receptor signaling, in bowel tissue. To determine whether IL-27 acted directly on colonic epithelium, murine colonoids and primary intact colonic crypts were shown to be unresponsive to IL-27 *in vitro* and to lack detectable IL-27 receptors. On the other hand, macrophages, which are present in inflamed colon tissue, were responsive to IL-27 *in vitro*. IL-27 induced pSTAT1 in macrophages, the transcriptome indicated an IFN-like signature, and supernatants induced pSTAT1 in colonoids. IL-27 induced anti-viral activity in macrophages and MHC Class II induction. We conclude that the effects of mucosal delivery of IL-27 in murine IBD are in part based on the known effects of IL27 inducing immunosuppression of T cells mediated by IL-10. We also conclude that IL-27 has potent effects on macrophages in inflamed colon tissue, generating mediators that in turn act on colonic epithelium.

## Introduction

Inflammatory bowel disease (IBD), characterized by pathological immune reactions to the gut microbiome, is comprised of two general types, Crohn’s disease and ulcerative colitis. Deficiencies in the IL-10 pathway have long been known to increase patient risk for IBD (recently reviewed in (1)). One activity of IL-10 is immunosuppression, which could dampen host immune responses to enteric bacteria. IL-10 has also been shown to suppress intestinal permeability, which is thought to precede IBD symptoms (reviewed in (2)). Moreover, IL-10 has recently been shown to mediate diurnal permeability (3). The cytokine IL-27 is a potent inducer of IL-10 (4) but has been less studied than IL-10 itself in IBD. IL-27 has diverse effects on a variety of cell types, including supporting the differentiation of CD4+IL-10+ T cells (5); inhibiting Th2, innate lymphoid cell-2, and Th17 responses (6); and supporting the differentiation of Th1 cells (7). Both immunostimulatory and immunosuppressive effects of IL-27 have been demonstrated on antigen presenting cells. IL-27 stimulation of human monocytes resulted in increased expression of the proinflammatory cytokines TNF-α, IL-6, IP-10, MIP-1α, and MIP-1β (8). Furthermore, costimulation of human monocytes and the human monocytic cell line THP-1 with both IL-27 and bacterial lipopolysaccharide increased the production of IL-6, TNF-α, and IL-12p40 relative to lipopolysaccharide alone (9). Interestingly, Kalliolias et al. (10) observed that IL-27 suppressed the response of human macrophages to TNF-α and IL-1β by reducing cell surface expression of the receptors for these cytokines and for IL-1β, increasing expression of antagonist and decoy receptors. A low expressing IL-27 genotype was shown to be a risk factor for IBD (11–13), suggesting that IL-27 could act upstream of IL-10 in suppressing IBD. IL-27 delivered enterically by engineered food bacteria was shown to have therapeutic efficacy in mouse models of IBD (14, 15), and treatment was associated with induction of phosphorylation of STAT1 (pSTAT1) in intestinal tissues (15). While pSTAT1 is a mediator of signal transduction by the IL-27 receptor, it is also a mediator of other cytokine receptor signals, IFNγR for example. Thus, IL-27 could: 1) act directly on intestinal epithelium and induce phosphorylation of STAT1 or 2) act on other cell types in inflamed bowel tissue inducing pSTAT1, and possibly trigger production of secondary mediators that induced pSTAT1 in intestinal epithelium. In the present study we addressed the question of which intestinal cell types could respond to IL-27 directly or indirectly. Our approach employed murine colon organoids and culture of other cell types present in inflamed colon. We conclude that murine colonic epithelium does not respond directly to IL-27 but does respond secondarily to inflammatory mediators induced by IL-27 in macrophages.

## Results

**Figure 1:**
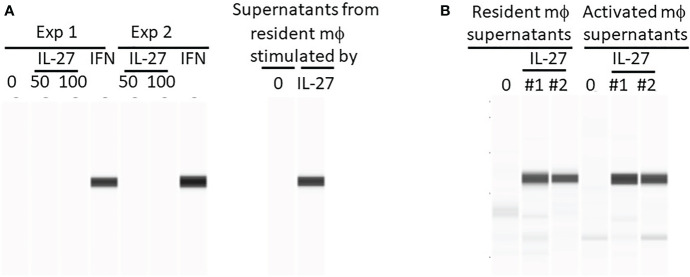
IL-27 does not directly induce Stat1 phosphorylation in murine colonoids but can induce indirectly *via* macrophage soluble factors. Colonoids were generated from C57BL/6 mouse colons and phosphorylation of Stat1 was detected by WES after the following stimulations: **(A)** Colonoids were either stimulated directly with IL-27 (50 or 100 ng/ml) or IFNγ (10 ng/ml) for 24h in two experiments, or with supernatants harvested from resident peritoneal macrophages that were stimulated with IL-27 for 24h. Two experiments are shown. **(B)** A comparison of IL-27-induced resident versus activated mϕ supernatants from two experiments (#1 and #2) in induction of Stat1 phosphorylation in colonoids.

Since we observed that pSTAT1 in colon tissue was induced by mucosal delivery of IL-27, we investigated the cell types involved in this response. To test whether IL-27 acted directly on colonic epithelium, we treated mouse colonoids with IL-27 *in vitro*. IL-27 did not induce pSTAT1 in colonoids (**Figure 1A**), compared with IFNγ as a positive control. IL-27 also had no effect on murine colon epithelial proliferation (data not shown). This appeared to conflict with the observation that IL-27 induced pSTAT1 in a human colorectal cancer cell line (16). We therefore tested the human colorectal cancer cell line HCT-116 and confirmed that, unlike our finding in mouse colonoids, IL-27 induced pSTAT1 (**Figure S1**). We considered the possibility that, *in vivo* in the mouse, IL-27 stimulated *via* the luminal surface of epithelial cells, but failed in culture because the receptor might be inaccessible due to apical polarity of colonoids. For example, perhaps the IL-27 receptor was expressed on the luminal surface of the colonoids that was inaccessible to IL-27 in medium, being sealed off from the exterior. However intact crypts that had not yet developed into spheroids also did not respond to IL-27 (**Figure S2**). In other studies (data not shown) we could not detect the IL-27 receptor in murine colon cells by western blot. The response of human colorectal cancer cell lines, but not mouse cells, may be due to a species difference, IL-27R being expressed on human (17) but not mouse colon epithelium.

We then considered the possibility that in murine IBD models, the pSTAT1 we had observed *in vivo* in IL-27-treated mice (15) was an IL-27 effect on cell types other than colonic epithelium. Because the inflamed colon is infiltrated with macrophages, it was possible that, *in vivo*, IL-27 had acted on macrophages inducing pSTAT1. Activated macrophages could then have produced factors (IFNγ for example) that subsequently induced pSTAT-1 in colonic epithelium. Resident or activated macrophages were stimulated with IL-27 *in vitro* and the supernatants were added to cultures of colonoids. As shown (**Figures 1A, B**), these macrophage supernatants were highly effective in inducing pSTAT1.

**Figure 2:**
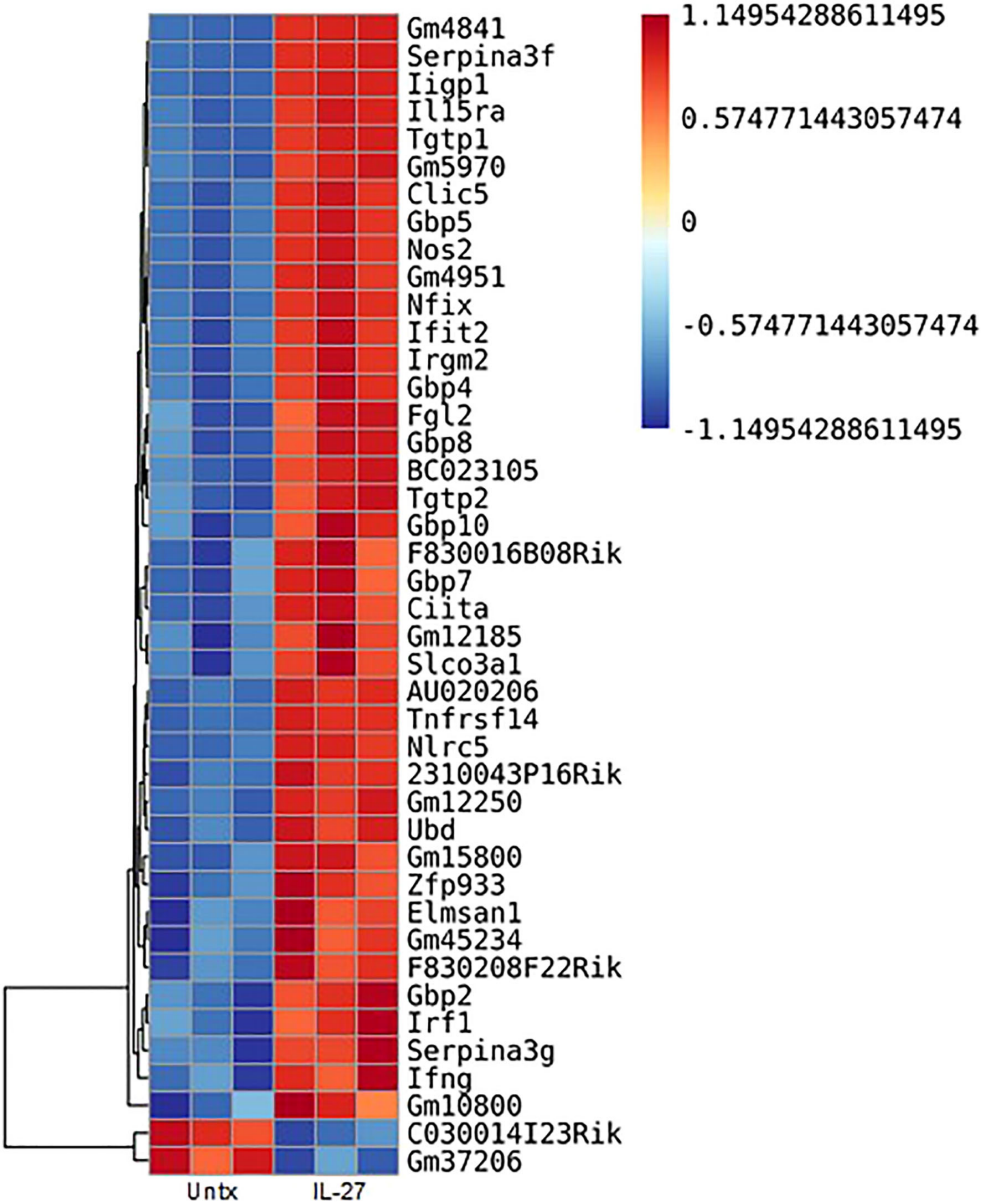
Transcripts in murine macrophages affected by IL-27 stimulation. Thioglycolate-induced peritoneal macrophages were cultured with 100 ng/ml IL-27 for 24h. Shown are 42 transcripts that were significantly changed by IL-27 stimulation. All data are representative of a minimum of at least three independent experiments. P<0.05. Graph depicts mean + SD.

**Figure 3:**
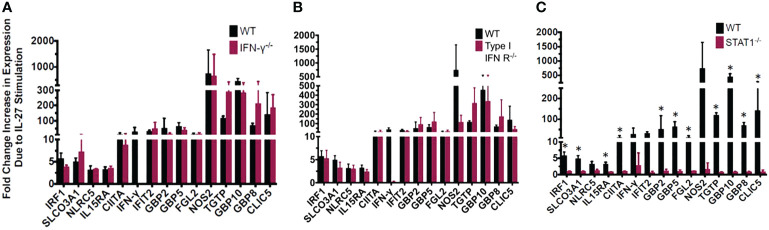
Most of the IL-27-induced “IFN signature” is not secondary to IFNs but is dependent on Stat1. The exception is induction of IFNγ which required both type I IFN and Stat1. IL-27-induced gene expression detected by real time RT-qPCR in wild type macrophages relative to those with genetic deletion of **(A)** IFNγ, **(B)** type I IFNR, or **(C)** STAT1. Graphs depict mean + SD. Data representative of 3 independent experiments. *p<0.05 by T-test of delta cycle thresholds of the gene expression in wildtype and the respective knockout strain.

**Figure 4:**
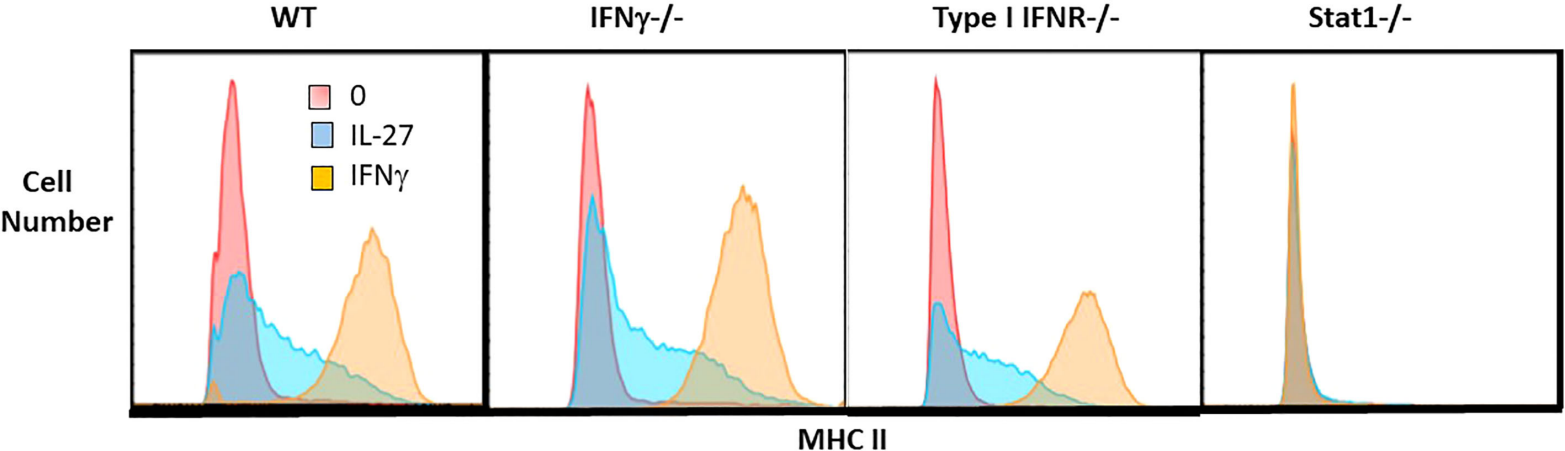
IL-27 induction of MHC II expression is independent of IFNγ or type I IFNR but dependent on Stat1. Single cell suspensions of macrophages from WT, IFNγ-/-, type I IFNR-/- or STAT1-/- mice were cultured for 48hr with IL-27 (100ng/ml) or IFNγ (10ng/ml) or control medium alone. Following culture, macrophages were detached with trypsin/EDTA and scraping. Non-specific Fc binding was blocked with anti-CD16/32, then cells were stained with rat anti-mouse I-A/I-E PE or PE rat IgG2b,k isotype control antibody (not shown). Cells were washed, fixed and analyzed by flow cytometry using FlowJo software.

**Figure 5:**
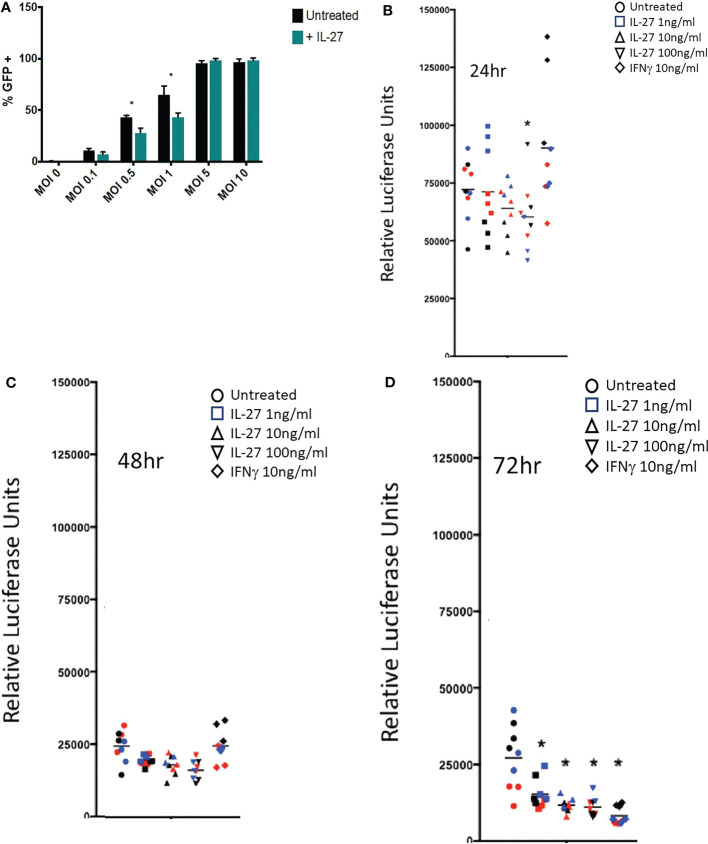
IL-27 treatment elicits antiviral activity against vaccinia and cytomegalovirus in murine macrophages. **(A)** Infection of resident macrophages with GFP-expressing vaccinia virus at six multiplicities of infection (MOI). IL-27 reduced viral infection of macrophages at 0.5 and 1 MOI. Graph depicts mean + SD of values from triplicate samples **(B-D)**. A luciferase-reporter mouse cytomegalovirus was used to infect macrophages in the presence of different IL-27 concentrations or IFNγ as a positive control. Supernatants were harvested at 24, 48 or 72h and used to infect a highly MCMV permissive fibroblast cell line as a measure of active virus release from infected macrophages. Luciferase activity in the supernatant of infected macrophages was evaluated at 18h post fibroblast infection. Data representative of three independent experiments are shown in three colors. *p<0.05 by two-way ANOVA.

IL-27 responses have been thoroughly studied in T cells, but macrophage responses have been less extensively examined. We therefore analyzed the transcriptome of thioglycolate elicited macrophages for IL-27 effects. As shown (**Figure 2**), 40 transcripts were increased, whereas 2 were decreased by IL-27 stimulation. **Table 1** lists 17 genes of particular interest because they are also associated with IFN induction, which may be due to the sharing of STAT1 in both signaling pathways. Thus, IL-27 could have IFN-like activities, as will be discussed further in the results seen in later **Figures 3**–**5**.

**Table 1:** RNA sequencing revealed significant increases in 17 genes of interest due to IL-27 stimulation for 24 hours in thioglycolate-induced M1 macrophages.

GENE	
***IFNG***	Interferon gamma*^#^
***CLIC5***	Chloride intracellular channel protein 5*^#^
***GBP2***	Guanylate-binding protein 2*^#^
***GBP5***	Guanylate-binding protein 5^*#^
***GBP7***	Guanylate-binding protein 7^*#^
***GPB8***	Guanylate-binding protein 8^*#^
***GPB10***	Guanylate-binding protein 10^*#^
***CIITA***	MHC class II transactivator^*#^
***NOS2***	Nitric oxide synthase, inducible^*#^
***NLRC5***	NLR family CARD domain containing 5^*#^
***GM12250/IRGB10***	Interferon-gamma-inducible p47 GTPase^*#^
***IRF1***	Interferon regulatory factor 1^*#^
***IL15RA***	Interleukin-15 receptor subunit alpha^*^
***IFIT2***	Interferon-induced protein with tetratricopeptide repeats 2^*^
***FGL2***	Fibroleukin^*^
***SLCO3A1***	Solute carrier organic anion transporter family member 3A1^*^
***TGTP1/2***	T-cell-specific guanine nucleotide triphosphate-binding protein ½^*#^

Many of these genes are associated with interferon signaling (*) or pathogen responses (#).

**Figure 6:**
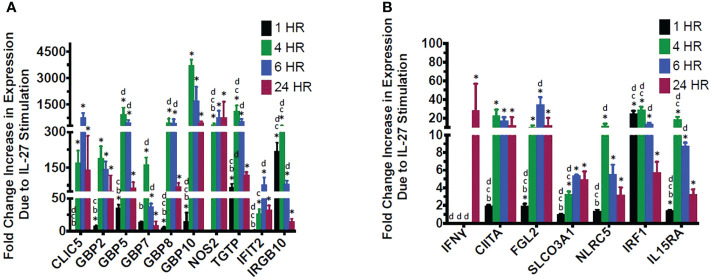
Validation of IL-27-induced transcripts in murine macrophages. Real time RT-qPCR validation of genes identified as upregulated by IL-27 by RNA sequencing of resident macrophages **(A, B)**. *p<0.05 by T-test of log-transformed delta cycle threshold values p<0.05 between gene expression timepoints by one-way ANOVA of log-transformed delta cycle threshold values. a=different versus expression at 1 hour. b=different versus expression at 4h. c=different versus expression at 6 h. d=different versus expression at 24h. Graphs depict mean + SD. Data representative of three independent experiments.

The 17 transcripts of interest were validated by real time PCR (**Figure 6**). Most transcripts required 4hr of induction, whereas IRGB10 and IRF1 were highly induced in 1hr. In contrast, IFNγ required 24hr of stimulation, which suggests intermediates are involved, as will be discussed further in results seen in later **Figures 3**–**5**. Although IL-27 induced NOS2 transcripts, we did not detect NO in supernatants (**Figure S3**) – this may explain why IL-27 lacked anti-salmonella activity (**Figure S4**) but showed anti-viral activity as will be discussed in results seen in later **Figure 5**.

We evaluated whether the “IFN-like” signature in IL-27-stimulated macrophages was due to IFNs themselves that could have been elicited by IL-27 stimulation. Macrophages from mice with deletion of IFNγ or type 1 IFNR were evaluated and compared with STAT1 deletion. As shown (**Figure 3**), all transcripts of interest were eliminated by STAT1 deletion. Neither of the IFN pathway deletions reduced the transcripts with one exception: IFNγ. As noted above, IFNγ induction was much delayed compared to the other transcripts (**Figure 6**) and from this data (**Figure 3**) appeared to be due to activation of the type 1 IFNR pathway, as well as STAT1.

To determine whether the transcript for the CIITA transcription factor (**Figure 6**) elicited the expected increase in class II MHC, macrophages were evaluated by flow cytometry. As shown, IL-27 increased the surface expression of class II MHC compared to unstimulated controls, although not to the level induced by IFNγ (**Figure 4**). This induction did not depend on either IFN pathway, but required STAT1, indicating it was likely a direct activity of IL-27 rather than an indirect effect *via* IFNs. Class II MHC induction could have a positive effect on antigen presentation in the adaptive immune response.

The IFN-like signature of IL-27 suggests it could have anti-viral activities. This was evaluated against vaccinia and cytomegalovirus infections in macrophages. As shown (**Figure 5A**), IL-27 reduced infection of macrophages at lower levels of infection with vaccinia virus. Activity of IL-27 against cytomegalovirus (**Figures 5B–D**) was determined by infecting macrophages with a luciferase-reporter mouse cytomegalovirus (MCMV) in the presence of IL-27 or IFNγ as a positive control. Infected supernatants were collected at the indicated time points and used to infect a highly MCMV permissive fibroblast cell line as a measure of active virus release from infected macrophages. IL-27, like IFNγ, reduced MCMV production in macrophages 72h post-infection (**Figure 5B**). Because IL-27 induced expression of both type I and II IFNs, it was possible that the anti-viral activity of IL-27 was indirect and was partly or entirely mediated secondarily *via* IFNs. We examined this possibility by deleting the type I IFNR because, as shown in **Figure 3**, IFNγ induction was downstream of the type I IFN signal; therefore deleting the type I signal would eliminate both Type I and II IFN effects. As shown in **Supplementary Figure S5**, IL-27 showed IFN-independent anti-CMV effects for 2 days after infection. After this period, the antiviral effects of IL-27 were IFN-dependent. Therefore IL-27 had both IFN-independent and IFN-dependent anti-viral activities in macrophages.

## Discussion

The therapeutic benefit of IL-27 we previously reported in mouse IBD models was associated with induction of pSTAT1 in intestinal tissue (15). To investigate the mechanism of this therapeutic benefit, we first examined the response of colonic epithelial cells to IL-27, and to our surprise, found no IL-27 responses in either murine colonoids or freshly isolated crypts. We presume this is attributable to a lack of detectable IL-27 receptors on these cells. We then examined responses to IL-27 of macrophages which are present in inflamed gut tissue. We observed that, in murine macrophages, IL-27 induced pSTAT1 and an IFN-like response that was STAT1-dependent and showed anti-viral activity. Supernatants from IL-27-activated macrophages elicited pSTAT1 in murine colonoids. These results suggested that *in vivo*, IL-27 did not act directly on murine colonic epithelium, but could act, in part, *via* macrophages in inflamed tissue. The full spectrum of IL-27 effects in the IBD context are complex and also require T cells and IL-10 to mediate its therapeutic benefit (14, 15, 18).

IL-27 effects on T cells have been studied extensively, whereas there are limited studies on macrophages. Kalliolias and Ivashkov (19) reported potent effects of IL-27 on human monocytes but little activity on bone marrow-derived murine macrophages by examining pSTAT1 and a limited number of target genes. Differences from our findings (**Figures 2**–**6**; **Table 1**) may be due to the cell populations studied (resident vs bone marrow-derived) and, in our study, the extensive panel of analyzed transcripts that revealed an IFN-like signature. Since IL-27R and IFNRs share STAT1 in their signaling pathway, it might be expected that IL-27 could elicit an IFN-like response. As in our studies, Vald´es-L´opez et al. (20) recently reported that IL-27 has IFN-like activities on human monocytes. Adding to the complexity of reported IL-27 effects, the induction of MHC class II expression on macrophages (**Figure 4**) suggests potential adjuvant properties of IL-27 in the adaptive immune response. Thus, while IL-27 offers promising therapeutic potential in IBD, there is much to learn about its mechanism.

## Methods

### Mice

Animal studies were approved by the NIH Animal Care and Use Committee. The following mouse strains were obtained from the Frederick National Laboratory Core

Breeding Specific Pathogen Free Facility: C57BL/6NCr, B6.129S7-Ifngtm1Ts/J,

B6.129S2-Ifnar1tm1Agt/Mmjax, and B6.129S(Cg)-Stat1tm1Dlv/J.

### Cell and tissue collection

Non-activated resident or thioglycolate-induced peritoneal macrophages were harvested from mice as previously described (21). Prior to all tissue harvests, mice were humanely euthanized with CO_2_ followed by cervical dislocation as a secondary method of euthanasia. Resident peritoneal macrophages were harvested from experimentally unmanipulated mice by lavage of the peritoneal cavity with 10 ml of sterile cold PBS (Corning). For thioglycolate induction of peritoneal macrophages, 1 ml of sterile 3% Brewer’s thioglycolate medium was injected into the peritoneum. Induced peritoneal macrophages were harvested five days post-injection by peritoneal lavage with 10 ml of sterile cold PBS. For all studies except for the Salmonella clearance assays, macrophages were isolated by immunomagnetic sorting with the Macrophage Isolation Kit (peritoneum, mouse) (Miltenyi Biotec). For Salmonella clearance assays, bone marrow-derived macrophages were plated and differentiated for five days with M-CSF. Macrophages were selected by plate adherence. For colon organoid preparation, colons were harvested following humane euthanasia described above, flushed with sterile cold PBS to remove feces, incised longitudinally, and placed in sterile cold PBS on ice until processed.

### Cell culture

For RNA-sequencing, gene expression, protein expression, or flow cytometry experiments, non-activated or thioglycolate-induced macrophages were cultured with 100 ng/ml IL-27 (R&D Systems) or 10 ng/ml IFNγ for 1, 4, 6, 24, or 48 hours in DMEM cell culture media (Corning) containing 1% penicillin/streptomycin (Corning) and 10% fetal bovine serum. For co-culture experiments, approximately 1 x 10^6^ macrophages were plated at the bottom of 24 well plates. Colonoids were prepared as described below and seeded in Matrigel within Transwell inserts (VWR). Co-culture experiments were performed in DMEM/F12 media (Corning) with 10% fetal bovine serum, 1% bovine serum albumin, 100 U/ml penicillin, 100 μg/ml streptomycin, 2mM GlutaMAX (Gibco), and 10 mM hepes.

### Flow cytometry

Single cell suspensions of macrophages were taken immediately after harvest or following culture by removal of the adherent macrophages by a 2 minute incubation at 37°C in 0.05% trypsin/0.53 mM EDTA (Corning) followed by gentle mechanical lifting from the plate with a sterile cell scraper. Cells were then washed with PBS and nonspecific binding was blocked by a 10 minute incubation at 4°C with anti-mouse CD16/32 antibody (TruStain FcX, BioLegend). Cells were again washed with PBS and then incubated for 20 minutes at 4°C with the fluorochrome conjugated antibody of choice for that experiment: rat anti-mouse F4/80 APC (BD Biosciences clone T45-2342), APC rat IgG2a,k isotype control antibody (BD Biosciences), hamster anti-mouse CD11c PE (BD Pharmingen clone HL3), PE Armenian hamster IgG1,l2 isotype control antibody (BD Pharmingen), rat anti-mouse I-A/I-E PE (BD Pharmingen clone M5/114.15.2), or PE rat IgG2b,k isotype control antibody (BD Pharmingen). Cells were then washed once more in PBS and resuspended in 1% paraformaldehyde for analysis by the NIH Cancer and Inflammation Program Flow Cytometry Core. Data were analyzed with FlowJo software.

### Gene expression analysis

RNA was extracted from macrophages or colonoids with either the Qiagen RNeasy Mini or Micro kit or TRIzol reagent (Invitrogen) as per the manufacturer’s instructions. RNA was then quantified and evaluated for purity and contamination via Nanodrop Spectrophotometer (Thermo Scientific). RNA for RNA sequencing was evaluated by both Nanodrop and Agilent Bioanalyzer. RNA was reverse transcribed to cDNA with the High-Capacity cDNA Reverse Transcription Kit (Applied Biosystems) as per the manufacturer’s protocol. No reverse transcriptase controls were prepared by following the same cDNA transcription protocol with the omission of the reverse transcriptase. For real time RT-qPCR, Taqman gene expression assay primer/probe sets for the genes of interest and TaqMan

Universal PCR Master Mix were purchased from ThermoFisher. Samples were analyzed via Applied Biosystems 7300 Real-Time PCR System. RNA sequencing was performed by the NCI

Frederick Advanced Technology Research Facility on an Illumina HiSeq2500 instrument.

### Viral infection assays

IL-27 anti-viral activity was tested against vaccinia virus and mouse cytomegalovirus (MCMV) infection. Resident peritoneal macrophages were treated overnight with 100 ng/ml IL-27 and then infected with a GFP-expressing vaccinia virus for 6hrs and infection was measured by flow cytometry. For MCMV experiments, resident peritoneal macrophages were first treated with 1, 10, or 100 ng/ml of IL-27 or 10 ng/ml of IFNγ for 6hrs. Then, media with the cytokines was removed, and macrophages were infected with MCMV-3DR, a gaussia luciferase-expressing MCMV (kindly provided by Martin Messerle, Hannover Medical School), at moi=0.1. Supernatants from these macrophage cultures were collected at 24, 48, and 72hrs post-infection and used to infect M2-10B4 fibroblasts. Luciferase activity in the supernatant of infected fibroblasts was determined 18 hr post-infection using the BioLux Gaussia Luciferase kit (New England Biolabs) and a Mithras LB 940 plate reader (Berthold).

### Bacterial infection assays

Bone marrow derived macrophages were either pretreated for 24 hours or treated at the time of Salmonella infection with 100 ng/ml IL-27. Cells were infected with a MOI of 5-10 mCherry-expressing Salmonella enterica serovar Typhimurium strain SL1344 and monitored for replication via fluorescence with an IncuCyte Live-Cell Analysis System (Sartorius) for 24hrs.

### Nitric oxide quantification

Nitrite as an indicator of nitric oxide was measured in cell culture supernatants *via* a Griess Reagent Kit (ThermoFisher) according to the manufacturer’s protocol.

### Protein analysis

Non-secreted proteins were evaluated by capillary western blot on the Protein Simple Wes instrument as per the manufacturer’s protocol. Prior to analysis, cells were lysed in RIPA lysis buffer (ThermoFisher) for 10 minutes on ice. Samples were then centrifuged for 5 minutes at 2500 rpm, and protein samples were removed to another tube for freezing at -20°C for later analysis. A total of 4 μg of protein was analyzed for each target. The following antibodies were purchased from Cell Signaling Technologies for use in the Wes instrument: Cleaved Caspase-3 (Asp175) Rabbit Antibody #9661 (used at 1:50 dilution), Phospho-STAT1 (Tyr701) (58D6) Rabbit mAb #9167 (used at 1:50 dilution), Stat1 Rabbit Antibody #9172 (used at 1:500 dilution), Caspase-3 Antibody #9662 (used at 1:50 dilution).

### Colon organoid culture

Colon organoids (colonoids) were derived based on a previously published protocol (22). Longitudinally incised colons flushed free of feces were washed twice in cold sterile PBS. The colons were then laid flat with the mucosal surface facing upward and the mucus layer and luminal epithelium was gently scraped away with a sterile glass slide. The colons were then washed again, cut into approximately 1-2 mm pieces, and washed another 5-10 times until the supernatant was clear. Colon pieces were then incubated in 2 mM EDTA for 30 minutes at 4°C. The supernatant was removed and replaced with PBS, the colon pieces were shaken by hand for 5 minutes, and the supernatant was removed. The colon pieces were resuspended in PBS and pipetted repeatedly with a seropipette to further mechanically loosen the intact crypts. This procedure was repeated approximately 5-6 times, with collection of the supernatant each time. The supernatant was filtered with a 100 μm cell strainer and centrifuged for 5 minutes at 300 G. The crypts were then resuspended in DMEM/F12 (Corning) culture medium containing 1% penicillin/streptomycin (Corning) and 100 μg/ml Primocin (Invivogen). The crypts were washed six times in this media with centrifuging for 2 minutes at 200 G to remove any contaminating single cells. Crypts were resuspended in Matrigel (Corning) and plated in either standard or Transwell (VWR) 24 well plates (50 μl of Matrigel/crypt mixture per well; approximately 750 crypts per well). Crypts were then overlain with the following culture media (complete media): DMEM/F12 media (Corning) containing 1% bovine serum albumin, 10% fetal bovine serum, 100 U/ml penicillin, 100 μg/ml streptomycin, 2mM GlutaMAX (Gibco), 10 mM hepes, 1X N2 and B27 supplements (Gibco), 1 mM N-acetylcysteine (Sigma), 50 ng/ml epidermal growth factor (ThermoFisher Scientific), 100 ng/ml Noggin (Peprotech), 1 μg/ml R-spondin (Peprotech), 100 ng/ml Wnt3a (R&D Biosystems), 10 mM nicotinamide (Sigma), 500 nMA83-01 (Tocris), 10 nM prostaglandin E2 (Sigma), 10 nM [Leu-15]-gastrin-1 (Sigma), 10μM SB202190 (Sigma), 2.5 μM thiazovivin (Stemgent), and 100 μg/ml Primocin

(Invivogen). 1μM Jagged-1 peptide (Anaspec) was embedded with crypts in Matrigel. When cultures were initiated, 2.5 μM CHIR99021 (Stemgent) and 10 μM Y-27632 dihydrochloride were included in media but were discontinued thereafter. Media was changed every other day. For DAMP/PAMP response assays, colonoids were co-cultured for 24 hours with macrophages +/- IL-27, Transwell inserts were transferred to new plates containing fresh either plain or complete colonoid media, and colonoids were stimulated with one of the following: 100 ng/ml LPS (Sigma), 100 μg/ml poly I:C (Tocris), or 100 pg/ml IL-1a (R&D Systems).

## Data availability statement

The original contributions presented in the study are publicly available. This data can be found here: NCBI GEO, GSE229280.

## Ethics statement

The animal study was reviewed and approved by National Cancer Institute at Frederick Animal Care and Use Committee.

## Author contributions

CA conducted experiments and wrote sections of the manuscript, JH, SP, TS, CM, and LR conducted experiments and analyzed data, MC analyzed data, MM, HH, OS-M, DW, HY, DM, and WL supervised experiments, SD provided direction and editing. All authors contributed to the article and approved the submitted version.

## Supplementary material

Supplementary Figure 1 IL-27 induced phosphorylation of STAT1 in human HCT-116 cells. Bands detected by capillary western blot for STAT1 and pSTAT1. Two experiments are shown.

Supplementary Figure 2 IL-27 does not induce pSTAT1 in intestinal murine crypts. Freshly isolated intestinal crypts were treated with 100 ng/ml IL-27. HepG2 cells, are shown as a positive control. Capillary western blot was used to detect STAT1 and pSTAT1. Two experiments are shown.

Supplementary Figure 3 IL-27 does not induce NO production by murine macrophages. Detection of nitrite was measured as an indicator of nitric oxide production in macrophages activated with IL-27 +/- LPS. IFNγ + LPS is shown as a positive control. Triplicate measurements are shown at 36 and 48h.

Supplementary Figure 4 IL-27 inhibits macrophage clearance of Salmonella. Macrophages infected with mCherry-expressing Salmonella enterica serovar Typhimurium strain SL1344 +/- treatment with IL-27 24h prior to or at time of infection.

Supplementary Figure 5 IL-27 reduced active viral particles acutely but required IL-27-induced interferon production for sustained viral inhibition. Mouse cytomegalovirus was used to infect type I. IFNR-/- macrophages in the presence of different IL-27 concentrations or IFNγ. Supernatants were harvested at 24, 48 or 72hrs. Active viral particles were determined by subsequent viral infection of susceptible fibroblasts as detected by luciferase read at 18hrs post fibroblast infection with infected type I IFNR-/- macrophage supernatants. Data representative of two independent experiments. *p<0.05 by T-test between treated and untreated controls. Each data point color represents triplicates from each independent experiment.
