# Supplementary material for: IL-27 induces an IFN-like signature in murine macrophages which in turn modulate colonic epithelium
Source: Front Immunol. 2023 Apr 20;14:1021824. doi: 10.3389/fimmu.2023.1021824 (PMC10157156; doi:10.3389/fimmu.2023.1021824)
Supplement: Supplementary Figure 1 — IL-27 induced phosphorylation of STAT1 in human HCT-116 cells. Bands detected by capillary western blot for STAT1 and pSTAT1. Two experiments are shown. [file Image_1.pdf]

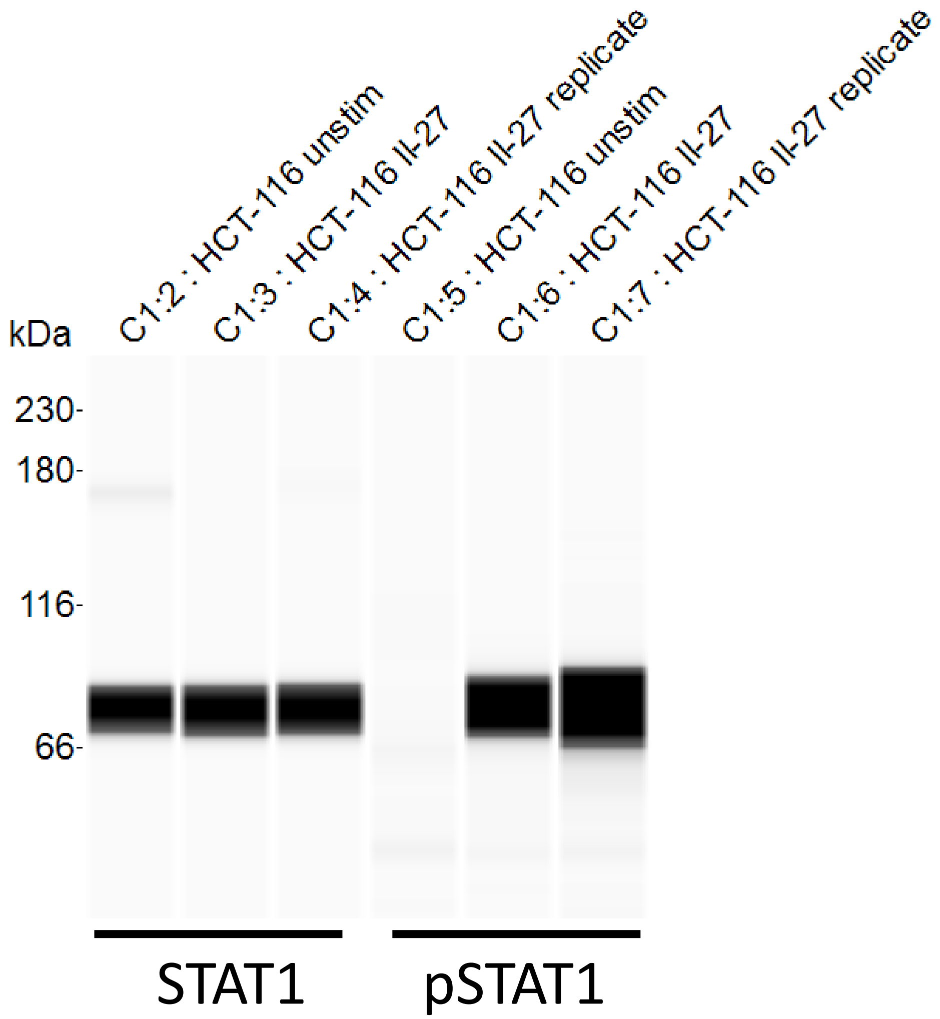

Fig.S1. IL-27 induced phosphorylation of STAT1 in human HCT-116 cells . Bands detected by capillary western blot for STAT1 and pSTAT1. Two experiments are shown.
