# Supplementary material for: IL-27 induces an IFN-like signature in murine macrophages which in turn modulate colonic epithelium
Source: Front Immunol. 2023 Apr 20;14:1021824. doi: 10.3389/fimmu.2023.1021824 (PMC10157156; doi:10.3389/fimmu.2023.1021824)
Supplement: Supplementary Figure 2 — IL-27 does not induce pSTAT1 in intestinal murine crypts. Freshly isolated intestinal crypts were treated with 100 ng/ml IL-27. HepG2 cells, are shown as a positive control. Capillary western blot was used to detect STAT1 and pSTAT1. Two experiments are shown. [file Image_2.pdf]

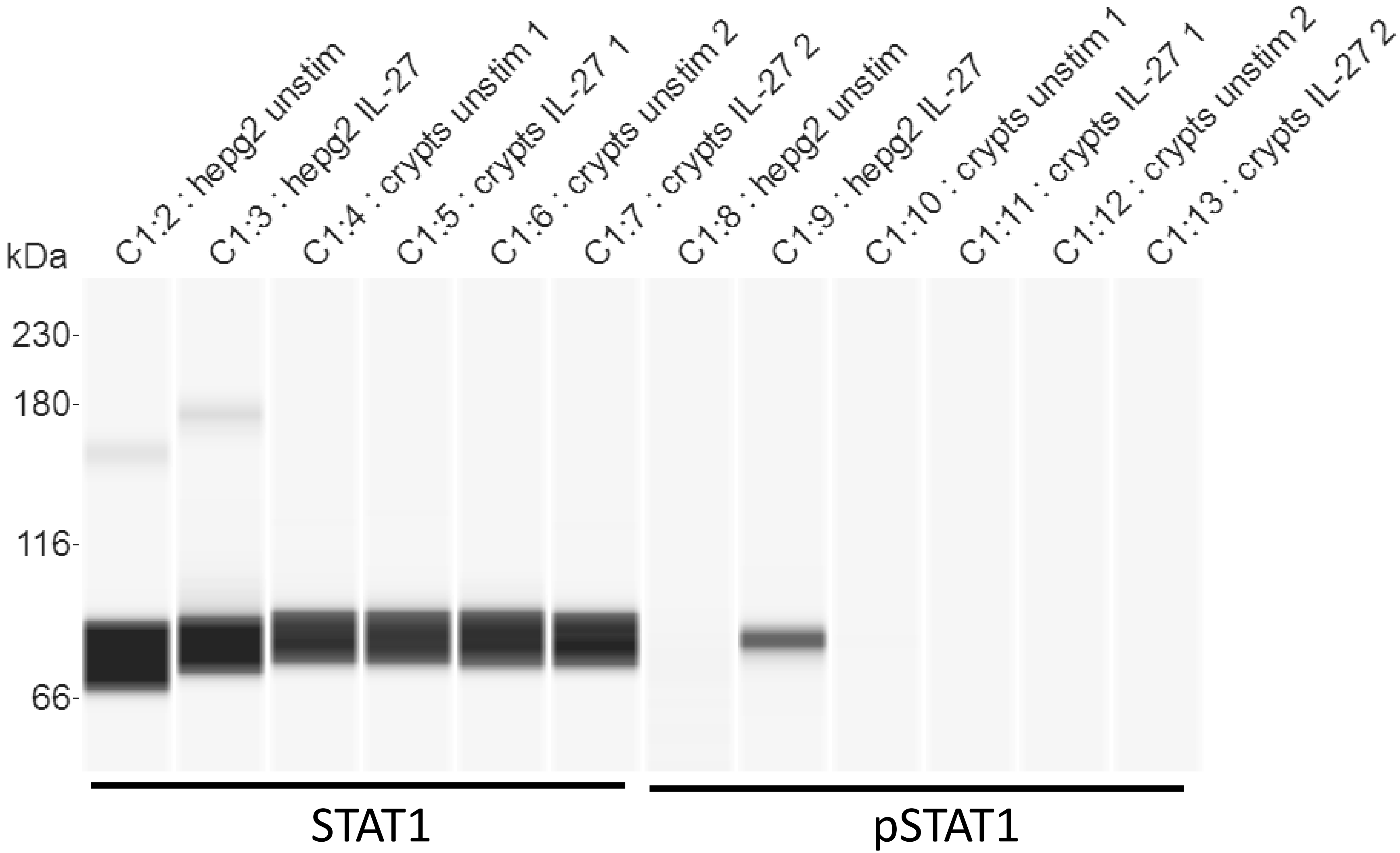

Fig.S2. IL-27 does not induce pSTAT1 in intestinal murine crypts. Freshly isolated intestinal crypts were treated with 100 ng/ml IL-27. HepG2 cells, are shown as a positive control. Capillary western blot was used to detect STAT1 and pSTAT1. Two experiments are shown.
