# Supplementary material for: IL-27 induces an IFN-like signature in murine macrophages which in turn modulate colonic epithelium
Source: Front Immunol. 2023 Apr 20;14:1021824. doi: 10.3389/fimmu.2023.1021824 (PMC10157156; doi:10.3389/fimmu.2023.1021824)
Supplement: Supplementary Figure 3 — IL-27 does not induce NO production by murine macrophages. Detection of nitrite was measured as an indicator of nitric oxide production in macrophages activated with IL-27 +/- LPS. IFNγ + LPS is shown as a positive control. Triplicate measurements are shown at 36 and 48h. [file Image_3.pdf]

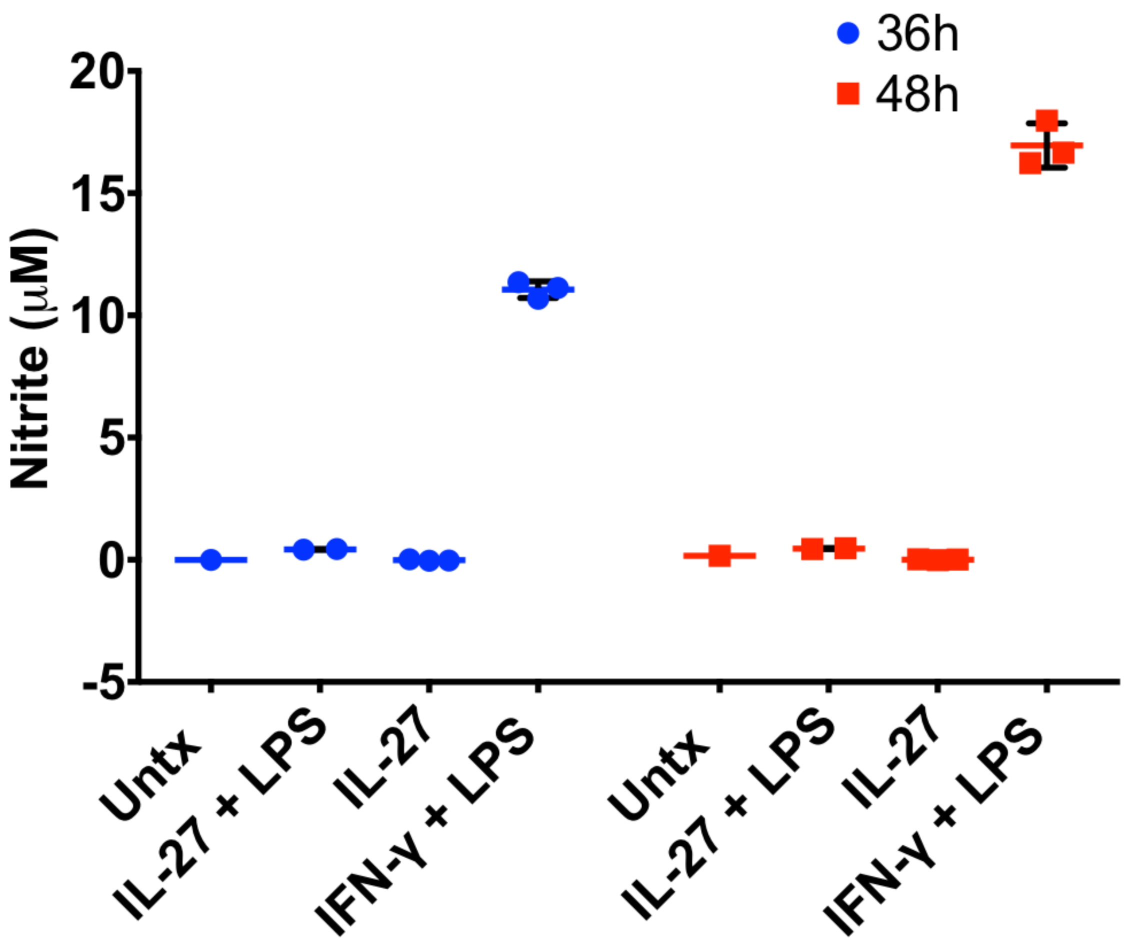

Fig.S3. IL-27 does not induce NO production by murine macrophages. Detection of nitrite was measured as an indicator of nitric oxide production in macrophages activated with IL-27 +/- LPS. IFN-g + LPS is shown as a positive control. Triplicate measurements are shown at 36 and 48h.
