# Supplementary material for: IL-27 induces an IFN-like signature in murine macrophages which in turn modulate colonic epithelium
Source: Front Immunol. 2023 Apr 20;14:1021824. doi: 10.3389/fimmu.2023.1021824 (PMC10157156; doi:10.3389/fimmu.2023.1021824)
Supplement: Supplementary Figure 4 — IL-27 inhibits macrophage clearance of Salmonella. Macrophages infected with mCherry-expressing Salmonella enterica serovar Typhimurium strain SL1344 +/- treatment with IL-27 24h prior to or at time of infection. [file Image_4.pdf]

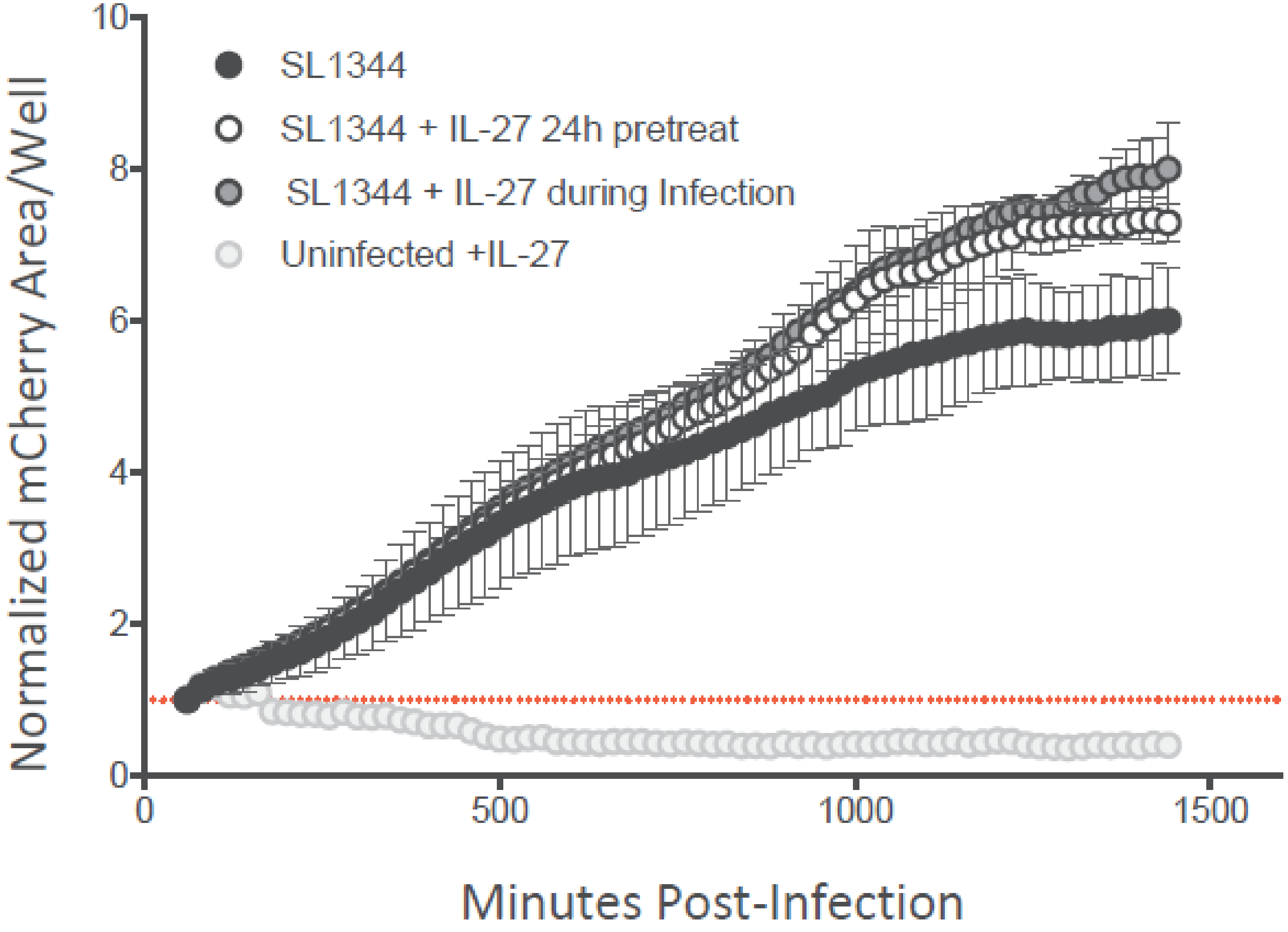

Fig.S4. IL-27 inhibits macrophage clearance of *Salmonella*. Macrophages infected with mCherry-expressing *Salmonella enterica* serovar Typhimurium strain SL1344 +/- treatment with IL-27 24h prior to or at time of infection.
