# Supplementary figures and images for: IL-27 induces an IFN-like signature in murine macrophages which in turn modulate colonic epithelium
Source: Front Immunol. 2023 Apr 20;14:1021824. doi: 10.3389/fimmu.2023.1021824 (PMC10157156; doi:10.3389/fimmu.2023.1021824)

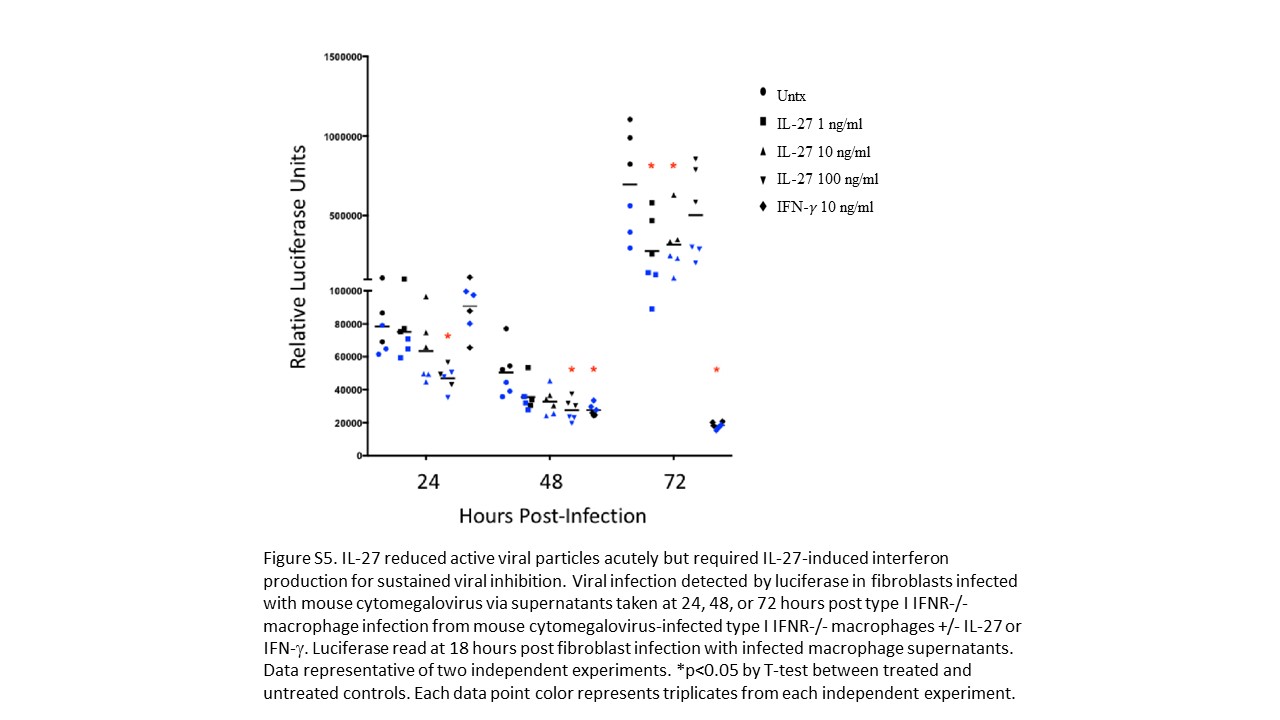

Supplement: Supplementary Figure 5 — IL-27 reduced active viral particles acutely but required IL-27-induced interferon production for sustained viral inhibition. Mouse cytomegalovirus was used to infect type I. IFNR-/- macrophages in the presence of different IL-27 concentrations or IFNγ. Supernatants were harvested at 24, 48 or 72hrs. Active viral particles were determined by subsequent viral infection of susceptible fibroblasts as detected by luciferase read at 18hrs post fibroblast infection with infected type I IFNR-/- macrophage supernatants. Data representative of two independent experiments. *p<0.05 by T-test between treated and untreated controls. Each data point color represents triplicates from each independent experiment. [file Image_5.jpg]
